# Evaluating Traditional Chinese Medicine and Herbal Products for the Treatment of Gestational Diabetes Mellitus

**DOI:** 10.1155/2019/9182595

**Published:** 2019-12-03

**Authors:** Yang Xin Zi Xu, Shengyan Xi, Xiaoyan Qian

**Affiliations:** ^1^Physiology and Pathophysiology, University of Manitoba, Winnipeg, Canada; ^2^Department of Traditional Chinese Medicine, School of Medicine, Xiamen University, Xiamen, Fujian Province, China

## Abstract

Gestational diabetes mellitus is the most common metabolic disorder during pregnancy with health consequences for both lives during and after pregnancy. Studies found that many pregnant women turn to complementary and alternative medicine for health maintenance or symptom relief, such as herbal medicine and acupuncture from traditional Chinese medicine. With the growing popularity of traditional Chinese medicine, we conducted a systemic search in PubMed, Web of Science, and Embase databases on research studies that investigated traditional Chinese medicine during pregnancy. The resultant hits were further searched in relation to all diabetes mellitus. In total, we found three major herbal medicine/herbal products that were associated with glycemic control in gestational diabetes, including Zuo Gui Wan, red raspberry leaves, and Orthosiphon stamineus. We further reviewed them and their relatives in relation to type 2 diabetes mellitus and found more evidence of metabolic benefits. None of the herbal medicine and products examined reported toxicity in the experimental models. Overall, treatments of gestational diabetes by western or alternative interventions are grossly understudied. It is critical to have a standardized protocol when evaluating efficacy of herbal medicine and produce quality results for women and their health-care providers to make informed treatment decisions.

## 1. Introduction

Pregnancy is a unique period during a female's life characterized by a series of physiological and metabolic changes. The maternal metabolism starts out with early anabolic accumulation of adipose tissue and progresses to late-stage catabolic release to accommodate feto-placental needs from organogenesis to fetal maturation and growth [1]. In the last trimester, anti-insulin hormones, growth factors, and cytokines released by the placenta produce an insulin resistance-like state, which is further exacerbated for those with excess weight, increased maternal age, polycystic ovarian syndrome, and family history of diabetes [2, 3]. Health issues that arise during this period put both the mother and the fetus at greater risk of complications during and after pregnancy.

Gestational diabetes mellitus (GDM) is the most common metabolic complication for pregnant women, which positively correlates with type 2 diabetes mellitus (T2DM) later in life [4]. In 2017, about 1 in 7 births is affected by GDM globally [5]. The prevalence of GDM is especially high among the Asian population [6]. In China, the most updated research found that on average, 14.8% of women develop GDM, and the risk increases significantly in older (26.7%) and overweight/obese women (30.3%) [7]. The continued increase of GDM posts heavy strain on the health-care system and calls for immediate action. GDM is often diagnosed between 24- and 28-week gestation (second or third trimester of pregnancy) and shares many pathological similarities with T2DM. Glucose is the primary fuel for the fetus and placenta. Under GDM, maternal insulin resistance increases the flux of glucose to the fetal circulation and contributes to increased fetal glucose uptake and growth [8]. During delivery, the mother may experience more respiratory distress, birth injuries, and cardiac abnormalities as well as preterm delivery. Babies born to GDM mothers are prone to congenital abnormalities such as macrosomia. While GDM often goes away after delivery, both the GDM mother and baby are now at an increased risk of developing T2DM later in life. Current treatments for GDM include diet, lifestyle intervention, and antihyperglycemic medications, and insulin is prescribed when the aforementioned fail to control blood glucose. In fact, insulin is one of the most commonly used prescription medications reported in pregnant women between 15 and 44 years of age [9]. It has been found that GDM women on insulin have progressive increments in insulin dependence as pregnancy advances [10]. Concerns still remain about the long-term safety of such treatment for the mother and child due to lack of quality research. A meta-analysis found poorly reported results and potential bias from randomized trials comparing treatments for GDM [11]. On the other hand, about half of pregnant women visit complementary and alternative medicine practitioner for pregnancy-related health conditions [12]. Women who visit their general practitioners and midwives more frequently are also more likely to visit acupuncturists for conditions such as gestational diabetes [12]. They are reportedly motivated by factors such as a sense of self-determination, pursue of natural and safe childbirth and a deeply personal and positive therapeutic experience [13]. Yet, there are extremely little research on both mainstream and alternative GDM treatments, which severely limits the ability for women and their health-care providers to make informed treatment decisions. It is also important to note that the placenta becomes thinner as gestation progresses and is permeable to certain drugs; and, the fetal liver has limited capacity to metabolize them [14]. One study suggests that metformin, the most prescribed antidiabetic drug, is weakly toxic towards embryonic stem cells, and should be prescribed with caution to pregnant women [15]. Therefore, it is absolutely critical to understand drug metabolism in adults as well as fetuses when treating GDM.

## 2. Traditional Chinese Medicine

Traditional Chinese Medicine (TCM) is a popular naturopathic medicine that advocates the balance of two opposing yet complementary forces: Yin and Yang, and the maintenance of Qi (the natural flow of energy). This branch of medicine presents a different point of view in understanding human physiology based on historical precedence and cumulative observations, which dates back to more than two millennia ago. TCM puts emphasis on modulating the body's own resistance against disorders and produces highly individualized treatment plan according to syndrome differentiation. TCM practitioners long ago have referred to diabetes mellitus as the Xiao-Ke diseases (wasting and thirst) and described its pathogenesis as the deficiency of Yin and stagnation of Qi, which lead to excess heat and dryness. *S*ymptoms recorded in *The Yellow Emperor's Classic of Internal Medicine* described diabetes mellitus as “three excess” and “one loss,” which correspond to excess in thirst, hunger and urination, and loss in weight [16]. The major organs affected are the lung, spleen, and kidney; therefore, TCM treatment for diabetes mellitus centers on ways to tonify Yin and resolve heat and dryness in these organs. According to TCM, pregnancy is the union of Yin and Yang, where Yin represents rest, accumulation, and storage, while Yang is required during birth. The spleen is considered the origin of fetal growth and development, and the kidneys store essence that nourishes the fetus [17]. Both organs are implicated in GDM.

Among the modern methods of TCM, herbal medicine is the more commonly accepted practice, and it is utilized throughout human history in many parts of the world. It is estimated that more than 80% of the world's population rely on herbal medicinal products as a source of primary healthcare [18]. Herbal medicine in TCM is prescribed as a carefully selected formula of plant products, including leaves, stems, flowers, roots, and seeds, each with many active components. Nowadays, the scope of herbal medicine has extended to include minerals and animal products. The goal is to maximize therapeutic effects and minimize toxicity. Decoction or steeping in ceramic containers is the most common form of dose preparation, which evenly draws out the therapeutic constituents of the herbs. Though TCM, herbal medicine prescriptions rely heavily on experience and observations and research efforts have been put in to unveil the pharmacological underpinnings. A prime example is demonstrated by the discovery of artemisinin from a Chinese herbal medicine compound, sweet wormwood (*Artemisia annua*) by the 2015 Nobel Prize winner, Youyou Tu. A Chinese pharmacologist in training, Tu's discovery is the perfect synthesis of historical treatises of Chinese herbs and modern scientific analysis and clinical approach.

Although TCM has enormous potential, philosophical and cultural differences and a lack of consistent and rigorous evidence-based studies still result in a general lack of acceptance by the western medical community. Multiple studies on the safety of Chinese herbal medicine during pregnancy was also inconclusive due to poor experimental stratifications [19–21]. In this review, we aim to identify available studies that specifically focus on herbal medicine treatment of GDM. A systematic literature search was conducted in PubMed, Web of Science, and Embase databases using different combinations of keywords, including gestational diabetes, traditional Chinese medicine, Chinese herbal medicine, antidiabetic, and hyperglycemia. The resultant hits were then further searched in relation to diabetes mellitus in general. With the growing popularity of complementary and alternative medicine worldwide, it is necessary to review and revise future approaches.

### 2.1. Zuo Gui Wan (Zuo Gui Pill)

Zuo Gui Wan is a Ming dynasty (1368 to 1644 A.D.) formula used to nourish Yin and tonify the kidney. The tablet form consists of a proprietary blend of 8 ingredients listed in Table 1. In a streptozotocin-induced rodent model of GDM, Zuo Gui Wan treatment reduced fasting blood glucose, body weight, total cholesterol, and serum insulin level of mice fed on high fat and sugar diet [22]. Furthermore, the same group found that when the offspring of Zuo Gui Wan-treated GDM mice were fed a high fat and sugar diet, they were protected against high levels of fasting plasma glucose, insulin, leptin, total cholesterol, and low-density lipoprotein [23]. These results suggest a cross-generational effect of Zuo Gui Wan on GDM mother and offspring through an unknown mechanism. A recent study found direct benefits of Zuo Gui Wan to fetal development and metabolism under high glucose stress when it was supplemented to cultured blastocysts [24]. Another study showed that Zuo Gui Wan facilitated ribosomal and mitochondrial functions in two-cell mouse embryonic cells during sugar metabolism, thus protecting them against cell death induced by glucose loading [25]. A related compound, Zuogui Jiangtang Jieyu Fang (with antidepressive property), also has well-documented glucose-lowering effect [26, 27]. Other than its antidiabetic property, Zuo Gui Wan is implicated in a wide range of disorders in reproduction [28, 29], bone metabolism [30–33], the immune system [34], and others that arise from the underlying diagnostic pattern according to TCM. Given its dual benefits for both the mother and fetus, Zuo Gui Wan may be a good therapeutic alternative or an augmentation to western medicine for GDM patients.

### 2.2. Rubus Idaeus (Red Raspberry Leaf)

*Rubus idaeus* belongs to the Rosaceae family, which is a diverse group of flowering plants that are used by many traditional medicine practitioners [34]. In one case study, researchers examined the consumption of red raspberry leaf tea by a pregnant woman with GDM and revealed properties of glycemic control of the herb [35]. This GDM patient experienced hypoglycemia after consumption of two servings of raspberry leaf tea for 3 days and resulted in a reduction in insulin requirements. The causal relationship was further supported by the patient's self-withdrawal and reintroduction of the herb. Metabolic exams and fetal surveillance revealed no abnormalities. While this isolated study sets precedence for *Rubus idaeus* in lowering blood glucose in humans, other identified benefits of the plant may help indirectly reduce GDM occurrence and severity. Chemical profiling of *Rubus idaeus* found the highest total phenolic and flavonoid contents in the leaves, which exhibit antioxidant activities and inhibit digestive enzymes, *α*-glucosidase, and *α*-amylase from producing monosaccharides [36]. Consumption of *Rubus idaeus* fruit in diabetic (db/db) mice reduced proinflammatory plasma interleukin 6 and may protect against diabetes-induced oxidative stress [37].

Other members of the same genus (listed in Table 2) also have documented hypoglycemic effect. For example, in rats fed a standard diet, *Rubus fruticosus* extract increased lipolysis of adipose tissue and enhanced insulin sensitivity, which was more pronounced in females [38]. In diabetic rats induced by either alloxan or streptozotocin, oral administration of *Rubus fruticosus* aqueous extract elicited hypoglycemic effects [39, 40]. The acute toxicity test found the lethal dose 50% (oral) for the aqueous extracts to be 8.1 g/kg, which makes the plant safe for consumption. However, several other studies could not find any glycemic properties of *Rubus fruticosus* using in vitro glucose absorption model and in diabetic mice [41, 42]. Preparation of the medicinal plant and the duration of treatment may have contributed to these inconsistencies. The Rubus plant documented in TCM is *Rubus chingii*, which functions to tonify the kidneys and preserve Qi. Ellagitannins from the unripe fruits of *Rubus chingii* has remarkable inhibitory activities against *α*-glucosidase and *α*-amylase [43]. Furthermore, the methanolic extract of *Rubus chingii* fruits was found to have enhanced inhibitory activities against protein tyrosine phosphatase 1B, a negative regulator of leptin and insulin signaling pathways [44]. On the other hand, the methanolic extract of *Rubus grandifolius* exhibits strong inhibition of glucosidases and prevents protein glycation with no cytotoxicity towards cultured cells [45]. A clinical study also supported the in vivo benefits of a North American relative, *Rubus occidentalis*, in controlling glycemia and vascular inflammation in prediabetic patients [46].

### 2.3. Orthosiphon stamineus

*Orthosiphon stamineus* is commonly known as the cat whisker and is the main ingredient of Java tea. In addition to its diuretic effect, a recent review also praised the antimicrobial, antioxidant, cytotoxic, and anti-inflammatory activities of *Orthosiphon stamineus* [47]. In both nonpregnant and pregnant diabetic rats, oral administration of *Orthosiphon stamineus* stimulated glucose-induced insulin secretion, which translated to an increase in ghrelin and glucagon-like peptide 1, and an overall lowering of glucose [48]. Pancreatic islets incubated with *Orthosiphon stamineus* also were significantly more sensitive to glucose-stimulated insulin release [48]. Urine metabolomics of diabetic rats treated with aqueous extract of *Orthosiphon stamineus* showed better regulation of the tricarboxylic acid cycle, glycolysis/gluconeogenesis, and lipid and amino acid metabolism [49]. From another study, the aqueous extracts (0.2-1.0 g/kg) significantly decreased plasma glucose concentration in a dose-dependent manner in both normal and diabetic rats [50]. The highest dose had comparable effect as the T2DM medication, glyburide. Plasma analysis revealed reduced triglyceride and elevated HDL-cholesterol concentration in the extract-treated diabetic rats, indicating an improved lipid profile [50]. Another chloroform extract from the plant also displayed antidiabetic effect through extrapancreatic mechanisms [51]. A developmental toxicity study was conducted on *Orthosiphon stamineus*; out of the 4 doses (250, 500, 1000, and 2000 mg/kg/day) tested, there was no clinical signs of maternal toxicity, weight gain, and prenatal growth retardation. The highest dose did increase anogenital distance due to possible androgenic effect [52].

## 3. Concluding Remarks

In this review, we sample the web for TCM and herbal medicine specifically aimed at treating GDM. Zuo Gui Wan, red raspberry tea, and *Orthosiphon stamineus* displayed promising effect in lowering glucose and alleviating the associated pathophysiology of GDM with minimum toxicity to the mother and fetus. These findings hold certain inspiration and clinical significance for the research and treatment of GDM. There are other herbal formulae used in Chinese clinics that aim to resolve specific pathology in GDM such as Qi deficiency and weakness in the spleen and stomach or as adjunctive therapy to insulin. For instance, Qiwei Baizhu powder has demonstrated effectiveness against GDM of spleen deficiency type and decreased postprandial blood glucose [53]. Huang Qi (Astragalus), another commonly used TCM herb, has shown to reduce blood lipids and enhance the antioxidative activity in conjunction with insulin treatment [54]. Further experimental studies would help strengthen these observations. In general, treatments for GDM by mainstream or alternative interventions are grossly understudied. Given the biggest concern for pregnant mothers is the safety of therapies, there has been no data on the long-term risk assessment of various antiglycemic formulations and strengths in human pregnancy. Comparison of the effectiveness between insulin and other interventions was also inconclusive due to poorly reported evidence [55]. As TCM gains global popularity and new medicinal products are constantly introduced into the market, public health concerns surrounding their safety are also increasingly recognized. While few complications have been reported from the use of herbal products as many constituents are regularly consumed, the holistic approach of herbal formula may cause possible interference with existing prescription or unknown and unwanted side effects with serious consequences. A few reviews have attempted to quantify the benefits of other TCM practices such as acupuncture and Qi Gong on diabetes. A randomized trial that looked at pregnancy outcomes of women with or without acupuncture treatment during their IVF treatment did not find any significant difference between occurrence of gestational diabetes and hypertensive disorder, or any of the parameter measured [56]. In a recent review examining the safety and effectiveness of acupuncture for T2DM, reviewers again did not find convincing evidence but indicated that acupuncture may be recommended to patients as a supplementary treatment [57]. Another review found that practice of Qi Gong produced an overall positive outlook on T2DM, but there was a large variation in styles and definitions of qigong of the studies reviewed [58]. In conclusion, the lack of knowledge and inconsistent data are the biggest hurdles in understanding the efficacy of TCM in a variety of disorders. Overall, it is hard to conclude a definitive benefit of TCM practice for GDM per se based on lack of direct studies.

## Figures and Tables

**Table 1:** The medicinal constituents of Zuo Gui Wan.

Pharmaceutical name	Chinese name (pin yin)	Chinese name (characters)	Common English name
Rehmanniae radix praeparata	Shu Di Huang	熟地黄	Prepared Rehmannia root
Rhizoma dioscoreae oppositae	Shan Yao	山药	Common yam rhizome
Fructus corni officinalis	Shan Zhu Yu	山茱萸	Asiatic cornelian cherry fruit
Cervi cornus colla	Lu Jiao Jiao	鹿角胶	Deer antler glue
Colla carapacis et plastri testudinis	Gui Jia Jiao	龟甲胶	Glue of tortoise shell
Fructus lycii chinensis	Gou Qi Zi	枸杞子	Barbary wolfberry fruit
Semen Cuscutae Chinensis	Tu Si Zi	菟丝子	Dodder seed
Radix Achyranthis Bidentatae	Niu Xi	牛膝	Two-toothed Achyranthes root

**Table 2:** List of plants in the Rubus genus with potential antidiabetic properties.

Scientific name	Chinese name (pin yin)	Chinese name (characters)	Common English name
*Rubus chingii Hu*	Zhang Ye Fu Pen Zi	掌叶覆盆子	Palmleaf raspberry fruit
*Rubus idaeus L.*	Fu Pen Zi	覆盆子	European red raspberry
*Rubus fruticosus L.*	Ou Zhou Hei Mei	欧洲黑莓	European blackberry
*Rubus grandifolius L.*	Ye Hei Mei	野黑莓	Wild blackberry
*Rubus occidentalis L.*	Hei Mao Mei	黑茅莓	Black raspberry
